# Epidemiology of adult congenital heart disease among the general population in Kuwait

**DOI:** 10.1002/clc.23569

**Published:** 2021-02-09

**Authors:** Hasan Ashkanani, Idrees Mohiyaldeen, Hazem ElShenawy, Muath Alanbaei

**Affiliations:** ^1^ Faculty of Medicine Kuwait University Kuwait Kuwait; ^2^ Department of Cardiology Chest Diseases Hospital Kuwait Kuwait; ^3^ Chest Diseases Hospital Kuwait Kuwait

**Keywords:** cardiology, congenital, Kuwait

## Abstract

**Background:**

Adult congenital heart disease (ACHD) is a highly underrepresented entity in medical literature, especially in the middle‐eastern region.

**Hypothesis:**

This study is the first to assess the prevalence of adult congenital heart disease among the population of Kuwait.

**Methods:**

After a retrospective register review of patients in Kuwait being followed up in the chest diseases hospital was conducted, patients who fit the inclusion criteria were enrolled in the study. Using the American College of Cardiology Task Force 1 of the 32nd Bethesda conference classification of the severity of ACHD, the patients were classified into those with simple, moderate, and complex congenital heart diseases. The age and gender of the patients, as well as the type repair performed, and the residual cardiac findings were recorded to assess the association between the complexity and residuals. Associations were assessed using STATA 15.

**Results:**

A total of 611 patients were evaluated over a period of 18 months. The youngest participant was 20 years of age, and the oldest participant was 88 years old. Male participants with moderate congenital heart disease class were more common in our study population. Patients with complex congenital heart disease have more residual cardiac lesion than the moderate or simple groups. Almost (70%) of patients with complex cardiac anomalies have undergone either partial or complete repair. The most prevalent cardiac defect was atrial septal defect (21.5%). Tetralogy of Fallot was the most prevalent defect in the moderate group, representing (13%) of the group. The most prevalent anomaly in the complex group was double outlet right ventricle (DORV) representing (15.38%).

**Conclusion:**

Adult Congenital heart disease is a growing entity of heart disease due to advanced repair techniques. This population requires registries to document cases and assign specialists for the management and care of this special group of patients.

**Highlights:**

- First database of adult congenital heart disease in Kuwait.
- The most prevalent heart defect was ASD in Kuwait.
- TOF was the most prevalent defect in the moderate group; and DORV was the most prevalent in the complex group.
- Patients with moderate ACHD tended to have a more complete repair than those in the complex group.

## 1 BACKGROUND

Adult congenital heart diseases, or ACHD, are defined as the persistence of any structural abnormality present at birth that involves the heart and/or great vessels beyond 16 years of age.^1^ Due to the continuous improvement of pediatric cardiology and cardiac surgery, the prevalence of adult congenital heart disease is on the rise.^2^, ^3^ However, the prevalence of ACHD is in most cases still lower than the incidence of congenital heart disease, or CHD, at birth because of case mortality, loss to follow‐up either due to spontaneously resolved CHD or asymptomatic surgically repaired CHD, and poor adherence to follow‐up schedules.^9^

It is estimated that the incidence of congenital heart disease is 8/1000 live births, and recent advances in pediatric open‐heart surgery and interventional cardiology now allow more than 85% of these patients to survive into adulthood. There are approximately 50 million ACHD patients world‐wide.^4^, ^5^ In addition, the prevalence of ACHD is expected to keep increasing until 2050 in projections.^6^ In a recent systematic review, the overall prevalence of ACHD is about 3 per 1000.^10^

There have been several excellent reports about the estimated prevalence of ACHD in Canada, UK, and US^7^; however, there is not much data on Asia and in particular on the gulf region. Therefore, our aim in this study was to assess the overall prevalence of adult CHD in Kuwait, using a register of patients being followed up in the chest diseases hospital in Kuwait data from 2006 to 2008. In addition, cases were classified based on severity into simple, moderate and complex congenital disease using the American college of cardiology task force 1 of the 32nd Bethesda conference classification.^8^

## 2 METHODS

This is a retrospective study, which was conducted after a retrospective register review of patients being followed up in the chest diseases hospital, Kuwait. After the register review was conducted, eligible candidates were enrolled in the study in order to assess the prevalence of adult congenital heart disease. Using the American College of Cardiology Task Force 1 of the 32nd Bethesda conference classification of the severity of ACHD, the patients were classified into those with simple, moderate, and complex congenital heart diseases. In addition, the age and gender of the patients, as well as the repairs performed and the residual cardiac findings were recorded in order to assess the association between the complexity and residuals. Associations of or with were assessed using STATA 15.

The study was approved by the Ethical Committee of Chest Diseases Hospital, Ministry of Health. It was performed in observation of the latest version of the declaration of Helsinki. The requirement of using an informed consent form was waived in this study due to the retrospective nature, as well as the complete anonymity of each included patient in the study sample. All the data was collected in a 2‐week period (from March 29 to April 14, 2019) on patients following up between 2006 to 2008 at the chest diseases hospital, Kuwait. A total of 611 samples were included during this period.

Data was entered, cleaned, and analyzed using the Statistical Package for the Social Sciences version 25 (IBM). Univariate analysis was performed to calculate percentages, frequencies, means, and standard deviations. Significant associations between dependent and independent categorical variables were tested using the Pearson chi‐square test. The binary logistic regression model was used to adjust the odds ratio for potential cofounding variables. Age, gender, and level of experience with internet use were the variables included as covariates in the model for adjustment as they were significantly associated with the score in the crude analysis. A *p* value of ≤.05 and 95% CI were considered to be the levels of significance.

## 3 RESULTS

**FIGURE 1:**
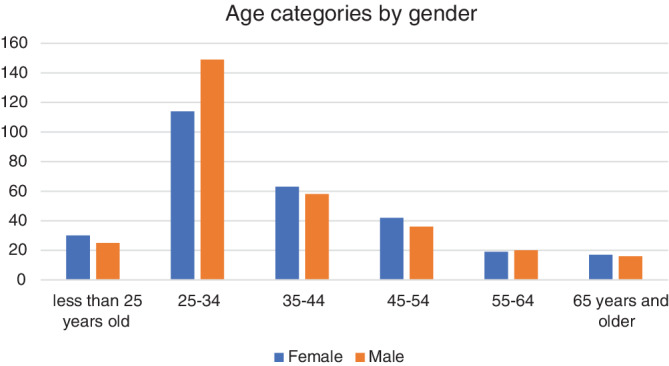
The distribution of different age groups by gender

In this study, a total of 611 patients were evaluated. Regarding the age distribution, more than half of the patients being in their twenties, whereas a minority of only 5.5% of the participants were older than 65 years old. The youngest participant was 20 years old while the oldest was 88 years old female with a secundum atrial septal defect (ASD), with the mean age being 38 years. Although male patients were numerically more in the moderate group, as well as in the overall number of cases, there was no statistically significant gender differences in all groups (Figure 1 and Table 1).

**TABLE 1:** Age categories by gender

Age group (years)	Male (%)	Female (%)	Total (%)
<25	25 (8.22%)	30 (10.52%)	56 (9.42%)
25‐34	149 (49.01)	114 (40.0%)	263 (44.58%)
35‐44	58 (19.08%)	63 (22.11%)	121 (20.51%)
45‐54	36 (11.84%)	42 (14.74%)	78 (13.22%)
55‐64	20 (6.58%)	19 (6.67%)	39 (6.61%)
65 or older	16 (5.26%)	17 (5.96%)	33 (5.59%)
Total	304 (100%)	285 (100%)	605 (100%)

*Note*: This tables displays the age distribution of adult congenital heart disease patients, stratified by gender. The highest proportion of the sample is in the age range of 25‐34 years.

In terms of the prevalence of ACHD in this study, the most prevalent cardiac defect overall was ASD including all its subtypes, as it constituted 21.5% of all cardiac defects. Also, of note, while ASD was the most common defect overall, upon stratifying the analysis by age, VSD was the most common diagnosis in the people aging 34‐year‐old or less, whereas ASD was still the most common diagnosis in all patients aged 35 years or more in the sample.

In the simple group, the most common ACHD was ASD, being diagnosed in 33% of the patients, followed by VSD, affecting 22.3% of the people enrolled in the study. The third most common diagnosis in the simple group was pulmonary stenosis, with 11.2% of the people enrolled in the study receiving it as a diagnosis.

Next, Tetralogy of Fallot was the most prevalent defect in the moderate group, representing 13.2% of the included sample. The second most prevalent defect was VSD when associated with other cardiac lesions, such as right ventricular outflow obstruction, aortic regurgitation, and coarctation of the aorta (CoA), representing 12.4%, followed by solitary CoA, which was diagnosed in 11.1% of this group.

The most prevalent anomaly in the complex group was double outlet right ventricle (DORV) representing 16.9% followed by congenitally corrected Transposition of Great Arteries (TGA), representing 12.9%. The third most prevalent complex ACHD was TOF at 10.4%, especially when it is associated with other cardiac anomalies.

In addition, when assessing those who underwent surgical and/or interventional repair, it was found that patients with moderate ACHD tended to have a more complete repair than those in the complex group (*p* < .001).

## 4 DISCUSSION

The necessity for a national registry documenting the cases of adult congenital heart diseases has become increasingly evident in recent years, with several countries already taking the initiative and starting their own registries, such as the CHALLENGE registry in Greece.^11^ These registries provide snapshots of the contemporary epidemiological data of ACHDs in a country, which is also one of the goals in our study in Kuwait. This study revealed that within 18 months, 611 patients have been registered, revealing for the first time, the epidemiology of this entity in Kuwait.

In this retrospective, the most prevalent subtype of ACHD was the atrial septal defect, constituting 21.5% of all adult congenital heart disease cases, followed by ventricular septal defects, and tetralogy of Fallot cases. In the registry that is used in Quebec, aortic valve dysfunction was the most prevalent diagnosis, followed by ASDs, as well as VSDs. In contrast, in the CONCOR registry used in the Netherlands, tetralogy of Fallot had the highest prevalence in the ACHD cases.^11^, ^12^ This difference could be explained by the difference in the number of participants, as well as genetic predispositions in each of the aforementioned samples.

Out of the total study population, 21% of the patients were classified into severe ACHD, a proportion that is quite higher than the Quebec study, which reported a proportion of 15% being diagnosed with severe adult congenital heart disease.^13^ The mean age of patients classified in severe subgroup in this study was 34 years, which is similar to the study conducted using the CHALLENGE registry in Greece, in which the mean age was 35 years.^11^ On the other hand, the Canadian registry reported a mean age of 25 years and in CONCOR 53 years in patients with severe ACHD.^12^, ^13^, ^14^ Of note, in registries with higher mean age at enrollment, patients with earlier attrition had not been registered.

Around 48% of the study population had undergone at least one reparative procedure. Although this proportion similar to the 50% reported result of the CHALLENGE study,^11^ it is significantly lower than the 76% reported in CONCOR registry,^12^ which may be due to the fact that the prevalence of defects differed in the studies.

The difference in gender distribution and diagnoses matched the available literature.^15^ Females had the majority of septal defects. On the other hand, tetralogy of Fallot and aortic arch abnormalities were more diagnosed in males. Men underwent more reparative surgical procedures than their female counterparts (Table 2).

**TABLE 2:** Distribution of repair by gender

Gender	Repaired (%)	Not repaired (%)	Total
Male	154 (49.36%)	158 (50.64%)	312 (100%)
Female	128 (45.39%)	165 (56%)	293 (100%)
Total	282	323	605

*Note*: This tables displays the proportion of repair distribution of adult congenital heart disease patients, stratified by gender. The highest proportion of the repair is in females, whereas more males with ACHD have not undergone reparative procedures.

Elderly (60+ years‐old) with ACHD constituted around 8% of the population sample. They mainly suffered from ASD in this study.^16^ These findings match the CHALLENGE registry used by Greece. In the CHALLENGE registry, the elderly accounted for 12% of the total population, suffering mainly from ASD. ASD was also shown to be the most prevalent subtype of ACHD in these patients in two other previous studies (Tables 3 and 4).^10^

**TABLE 3:** The top‐ranked diagnoses by complexity

Complexity	Male	Female	Overall
Simple	VSD (27.35%)	ASD (39.10%)	ASD (33.07%)
	ASD (26.50%)	VSD (17.29%)	VSD (22.31%)
	PS (13.68%)	PS (10.53%)	PS (11.95%)
Moderate	TOF (13.97%)	TOF (12.24%)	TOF (13.25%)
	COA (13.97%)	ASD (11.22%)	VSD (12.39%)
	VSD (13.24%)	VSD (11.22%) PS (11.22%)	COA (11.11%)
Severe	DORV (16.39%)	DORV (17.46%)	DORV (16.94%)
	CCTGA (13.11%)	TOF (12.70%)	CCTGA (12.90%)
	DTGA (11.48%)	CCTGA (12.70%)	TOF (10.48%)

*Note*: This tables displays the distribution of adult congenital heart disease, stratified by complexity. Adult congenital heart disease is classified according to complexity into simple, moderate, and severe categories.

**TABLE 4:** The top‐ranked diagnoses by age groups

Age groups	Top diagnosis	N (%)
<25 years	VSD	12 (21.43%)
25–34	VSD	42 (15.97%)
35–44	ASD	26 (21.49%)
45–54	ASD	22(28.21%)
55–64	ASD	10 (25.64%)
65 years or older	ASD	14 (42.42%)

*Note*: This tables displays the individual diagnoses of adult congenital heart disease, stratified by age.

The limitations of this study are identical to several registry‐based studies, including the fact that the full data of the study sample was not accessible; therefore, several areas, such as heart failure and arrhythmias, could not be defined in this study sample.

The ACHD‐affected population are a rapidly rising chip of society. National registries have become a necessity, as they are required to address their needs, as well as drive policy on organizing appropriate ACHD experts in centers with adequately trained staff, offering the highest quality of medical care to this portion of the community.

## 5 CONCLUSION

Adult congenital heart disease is a heterogeneous group of patients and because of the advanced repair techniques we are facing a surge in the number of patients who are recruited in ACHD clinic. It is intuitive that not all patients with simple lesions go for repair strategy, and some of them needed only conservative management and clinical follow‐up. The most prevalent heart defect was ASD with all its subtypes (secundum, primum, sinus venous, and unroofed coronary sinus). TOF was the most prevalent defect in the moderate group; and DORV was the most prevalent in the complex group.

Complex ACHD is a heterogeneous group of patients with multiple defects and different kinds of surgical and/or interventional repair that required a lot of investment in terms of diagnostic facilities, surgical, and interventional capabilities.

## CONFLICT OF INTEREST

The authors declare no conflict of interest.

## Supporting information


**DATA S1**: Supporting Information

**DATA S2**: Supporting Information

## Data Availability

Data is available in the supplementary material. It is also available with the corresponding author and can be provided upon request.
