# Supplementary material for: Epidemiology of adult congenital heart disease among the general population in Kuwait
Source: Clin Cardiol. 2021 Feb 9;44(4):526–30. doi: 10.1002/clc.23569 (PMC8027582; doi:10.1002/clc.23569)
Supplement: Supplementary file 1 — DATA S1: Supporting Information [file CLC-44-526-s001.pdf]

Dr.Hazem analysis

1- Age normality test

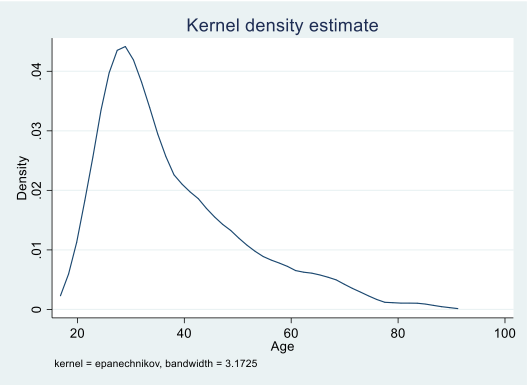

not normaly distributed

```
. kwallis age, by (classification)

Kruskal-Wallis equality-of-populations rank test
```

classi~n	Obs	Rank Sum
1	213	61564.00
2	238	70859.00
3	120	30883.00

```
chi-squared =     4.891 with 2 d.f.
probability =     0.0867

chi-squared with ties =     4.898 with 2 d.f.
probability =     0.0864
```

Using K-wallis test, There is no significant difference between ages for the 3 classifications as p= 0.086

2- Gender (male=1) vs classification (1=simple, 2=moderate, 3= complex)

```
. tab  classification male, col chi2
```

Key
frequency
column percentage

Classification	male 0	1	Total
1	117<br>40.77	104<br>34.10	221<br>37.33
2	105<br>36.59	140<br>45.90	245<br>41.39
3	65<br>22.65	61<br>20.00	126<br>21.28
Total	287<br>100.00	305<br>100.00	592<br>100.00

```
Pearson chi2(2) =   5.3493   Pr = 0.069
```

Using chi2 test, we find that p=0.69 (means that there is no significant difference between males and females according to classification.

3- (Repair=1 , not repaired=0) vs classification

```
. tab  classification repair , col chi2
```

Key
frequency
column percentage

Classification	repair 0	repair 1	Total
1	163<br>51.58	60<br>21.35	223<br>37.35
2	115<br>36.39	132<br>46.98	247<br>41.37
3	38<br>12.03	89<br>31.67	127<br>21.27
Total	316<br>100.00	281<br>100.00	597<br>100.00

Pearson chi2(2) = 67.4041   Pr = 0.000

Using chi2 shows $p<0.001$ meaning, there is significant difference in repair among classification.

```
. tabulate diagnosis classification
```

diagnosis	Classification 1	2	3	Total
ASD2	1	0	0	1
SUBAORTIC	1	0	0	1
ALCAPA	0	0	1	1
AR	0	1	0	1
AS	2	3	0	5
ASD	16	11	1	28
ASD1	4	2	0	6
ASD2	50	25	2	77
AVCD	0	1	0	1
AVM	0	0	1	1
AVSD	1	16	4	21
AVSD	0	1	0	1
BAV	12	13	1	26
CCTGA	0	1	15	16
COA	0	25	1	26
CORONARY FISTULA	1	0	0	1
CORONARY fistula	2	0	0	2
CoA	0	1	0	1
D TGA	0	0	1	1
DBL AORTIC ARCH	1	0	0	1
DCM	0	0	1	1
DEXTROCARDIA	0	1	0	1
DILV	0	0	6	6
DOLV	0	0	1	1
DORV	0	0	20	20
DTGA	0	0	7	7
EBSTIEN	0	7	0	7
ES	0	0	7	7
HOCM	5	3	1	9
INTER atrial	1	0	0	1
MARFAN	7	4	0	11
MR	2	0	0	2
MVP	13	5	1	19
PA	0	1	4	5
PA STENOSIS	0	0	1	1
PAPVC	0	0	1	1
PAVSD	0	0	1	1
PDA	9	10	2	21
PFO	12	0	0	12
PM	1	0	0	1
PR	1	1	0	2
PS	28	19	1	48
RVOTO	0	1	0	1
SUB AS	0	11	2	13
SUB aortic	0	1	0	1
SUPRA AS	0	0	1	1
SUPRAVALVULAR AS	0	1	0	1
SV ASD	1	11	4	16
TA	0	1	7	8
TAPVC	0	1	1	2
TGA	0	0	4	4
TOF	0	27	16	43
TOF	0	2	0	2
TRUNCUS	0	0	1	1
VSD	49	35	8	92
mitral	1	0	0	1
severe	0	1	0	1
single VENT	0	0	2	2
sub AS	0	1	0	1
subaortic	0	3	0	3
syndrome	1	0	0	1
vsd	1	0	0	1
Total	223	247	127	597
